# Superscattering of water waves

**DOI:** 10.1093/nsr/nwac255

**Published:** 2022-11-10

**Authors:** Zijian Qin, Chao Qian, Lian Shen, Xiaoping Wang, Ido Kaminer, Hongsheng Chen, Huaping Wang

**Affiliations:** Key Laboratory of Ocean Observation-Imaging Testbed of Zhejiang Province, Institute of Marine Electronics Engineering, Ocean College, Zhejiang University, Hangzhou 310058, China; ZJU-Hangzhou Global Science and Technology Innovation Center, Key Lab. of Advanced Micro/Nano Electronic Devices & Smart Systems of Zhejiang, Zhejiang University, Hangzhou 310027, China; ZJU-UIUC Institute, Interdisciplinary Center for Quantum Information, State Key Laboratory of Modern Optical Instrumentation, Zhejiang University, Hangzhou 310027, China; ZJU-Hangzhou Global Science and Technology Innovation Center, Key Lab. of Advanced Micro/Nano Electronic Devices & Smart Systems of Zhejiang, Zhejiang University, Hangzhou 310027, China; Jinhua Institute of Zhejiang University, Zhejiang University, Jinhua 321099, China; ZJU-UIUC Institute, Interdisciplinary Center for Quantum Information, State Key Laboratory of Modern Optical Instrumentation, Zhejiang University, Hangzhou 310027, China; ZJU-Hangzhou Global Science and Technology Innovation Center, Key Lab. of Advanced Micro/Nano Electronic Devices & Smart Systems of Zhejiang, Zhejiang University, Hangzhou 310027, China; Key Laboratory of Ocean Observation-Imaging Testbed of Zhejiang Province, Institute of Marine Electronics Engineering, Ocean College, Zhejiang University, Hangzhou 310058, China; Department of Electrical Engineering, Technion-Israel Institute of Technology, Haifa 32000, Israel; ZJU-UIUC Institute, Interdisciplinary Center for Quantum Information, State Key Laboratory of Modern Optical Instrumentation, Zhejiang University, Hangzhou 310027, China; ZJU-Hangzhou Global Science and Technology Innovation Center, Key Lab. of Advanced Micro/Nano Electronic Devices & Smart Systems of Zhejiang, Zhejiang University, Hangzhou 310027, China; Jinhua Institute of Zhejiang University, Zhejiang University, Jinhua 321099, China; Key Laboratory of Ocean Observation-Imaging Testbed of Zhejiang Province, Institute of Marine Electronics Engineering, Ocean College, Zhejiang University, Hangzhou 310058, China

**Keywords:** superscattering, water wave, metamaterials

## Abstract

Inspired by the concept of superscattering in optics, we for the first time theoretically predict and experimentally demonstrate the superscattering phenomenon in water waves. The subwavelength superscatterer is constructed by multi-layered concentric cylinders with an inhomogeneous depth profile. The superscatterer breaks the long-held single-channel scattering limit by several times and thus significantly enhances the total scattering strength. The underlying mechanism originates from the near degeneracy of the resonances of multiple channels. We fabricate the superscatterer prototype and experimentally measure the near-field patterns, which are consistent with theoretical prediction and numerical simulation. Our study opens a new avenue to strengthen water-wave scattering and deepen the understanding in water waves, which can be useful for ocean energy harvesting and harbor protection.

## Teaser

Enhancing water wave scattering can endow subwavelength entities with the ability to capture more energy from large areas, and water wave superscattering provide a new paradigm for achieving this goal and deepening the understanding of water wave propagation mechanism.

## INTRODUCTION

Understanding, utilizing and controlling water waves have been long pursued in both academia and industry, owning to their great potential in ocean energy harvesting, coastal protection, early warning of natural disasters and more. The realization of those goals has always used an enormous amount of financial and human resources for constructing energy converters and dam infrastructures. Recent advances in artificial structured materials have motivated scientists to revisit this community and transform the way we utilize water waves from passive defense to actively customizing the propagation manner of water waves [1–9]. Artificial structured materials in water waves actually originate from their successful counterparts in optics, electromagnetics and acoustics, termed as metamaterials [9–18]. Metamaterials refer to a family of periodic or aperiodic structures composed of subwavelength artificial atoms. During the past two decades, metamaterials have experienced a fast-paced development and given rise to a myriad of intriguing applications in terms of wavefront shaping, polarization conversion, optical display, acoustic absorption, storage and encryption. Similar physical principles have also been extensively penetrated into other types of waves. In water waves, the development pace is almost synchronous, starting from physical correspondences to application extensions, such as negative gravity [19,20], self-collimation [21–24], water-wave cloak [2,3,5,9,25–27] and concentrator [28]. We are witnessing that they serve as a driving force to revolutionize many transformative technologies and refresh the physical understanding of water waves.

Enhancing water-wave scattering can endow subwavelength entities with an ability to capture more energy from a large area. This notion has stimulated the development of a diverse set of applications, such as nano-generators driven by mechanical actuation, high-efficiency energy harvest and miniaturized breakwaters for harbor protection [29–32]. To this end, transformation optics (TO) offers a fresh theoretical paradigm, stemming from the formal invariance of Maxwell's equations [33,34]. For a given object, TO-based superscattering and illusion can arbitrarily boost the scattering cross section (SCS) to render it similar to that generated by a much larger object [26]. In physics, it is constructed by enclosing the given object with a carefully designed coating whose electrical permittivity and magnetic permeability are both spatially varying and anisotropic. Although the TO-based superscattering and illusion method is theoretically elegant, the experimental realization remains a great challenge due to the extreme constitutive parameters, i.e. highly inhomogeneous and anisotropic material parameters, as well as in water waves.

The other type of superscattering, induced by degenerate resonances, has recently been proposed and well recognized [13,35–41]. Despite the same terminology, its working principle is completely different from the TO-based superscattering. In the following, we refer by default to superscattering of the degenerate resonances type. The superscattering phenomenon was first revealed in a subwavelength multi-layered plasmonic-dielectric nanorod, where multiple plasmonic resonant modes were spectrally aligned [27]. The superscatterer can overcome the fundamental scattering limit of a subwavelength entity because its SCS is typically bound from a ‘cage’, namely the single-channel scattering limit. In a 2D scenario, this limit is $2\lambda /\pi $, where $\lambda $ is the wavelength (also applicable in water waves). Notably, such superscattering requires only inhomogeneous materials that easy to be fabricated. Subsequently, extensive theoretical works have been performed. In 2019, the superscattering was first observed in a microwave experiment, using a subwavelength metasurface-based multi-layer rod [42].

In this work, we for the first time theoretically predict and experimentally observe the superscattering phenomenon in water waves. The subwavelength superscatterer is constructed using multiple concentric cylinders with different water depths. By engineering an overlap of scattering peaks from different angular momentum channels, the total SCS can exceed the single-channel scattering limit by more than three times. In the experiment, we measure the near-field pattern of the fabricated superscatterer, which agrees well with theoretical prediction and numerical simulation. Furthermore, we explore the superscattering effect with different boundary conditions, water depths and frequencies. Our work provides an easy and low-cost way to enhance water-wave scattering and deepen our understanding of the fluctuation problem. We envision that the superscatterer may facilitate many applications, such as energy harvesting and near-shore wave protection [29–32].

## THEORY AND DESIGN

In nature, the fluctuations of water waves are various. Here we consider the water waves that propagate along the interface between air and water. Since their restoring force is mainly from gravity, they are collectively referred to as surface gravity waves. We extend the electromagnetic (EM) scattering theory to water waves in consideration of the similarity between the field equations of surface gravity waves and EM waves.

**Figure 1.**
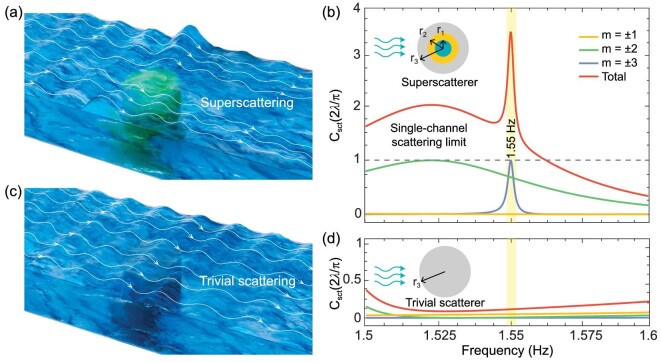
Schematic and working principle of water-wave superscattering. (a) The phenomenon of superscattering of water waves caused by superscatterer (green structure underneath) and the creation of a huge ‘shade’ behind the structure. The white curves at the water surface represent the Poynting vector lines. (c) Water waves flowing through a uniform cylinder (gray structure underneath) with the same size, termed as a trivial scatterer. When the water wave passes through the trivial scatterer, there is almost no change in the waveform. (b and d) Analytical total scattering cross section and the contribution from individual channels, caused by the superscatterer and the trivial scatterer. The inset shows the schematics of the two structures. The frequency range is from 1.5–1.6 Hz. At f = 1.55 Hz, the superscattering phenomenon occurs.

For conceptual simplicity, we consider linear, inviscid and irrotational water waves in an infinite range with constant depth *d*. The dispersion relationship of water wave is ${\omega }^2 = \ gktanh( {kd} ),\ $where $\omega $, *g* and $k = 2\pi /\lambda \ $represent the angular frequency, the acceleration of gravity and the wave number, respectively [43]. As illustrated in Fig. 1, we consider a 2D three-layer concentric superscatterer with heights of ${h}_1,\ {h}_2\ {\rm{and}}\ {h}_3$, and radii of ${r}_1,\ {r}_2\ {\rm{and}} {r}_3$ from the inside towards the outside. In other words, the superscatterer segments the whole region into four water depths of $d - {h}_1,d - {h}_2,d - {h}_3$ and *d*. We assume that the harmonic water waves propagate along the $+ x$ direction. The vertical displacement of the water surface $\eta $ is related to the potential $\varphi $, i.e. $\eta \ ( {{\boldsymbol{r}},t} ) = {\mathop{\rm Re}\nolimits} [ { - \frac{{i\omega }}{g}\varphi ( {\boldsymbol{r}} ){e}^{ - i\omega t}} ]\ $, where ${\boldsymbol{r\ }} = ( {x,y} )$ in the horizontal plane. $\varphi $ satisfies the 2D Helmholtz equation ${\nabla }^2\varphi {\rm{\ }} + {k}^2\varphi {\rm{\ \ \ }} = 0$. In the cylindrical coordinates with $( {\rho ,\phi ,z} )$, the incident plane wave can be represented by an infinite sum of cylindrical waves. In the outermost region, the total potential $\varphi $ can be expressed as:


(1) \begin{eqnarray*} {\varphi }_{total} &=& {\varphi }_0\ \mathop \sum \limits_{m=- \infty }^\infty \big( {i}^m{J}_m\left( {{k}_4\rho } \right){e}^{im\phi }\\ &&+\, {i}^m{S}_mH_m^{\left( 1 \right)}\left( {{k}_4\rho } \right){e}^{im\phi } \big), \end{eqnarray*}

where ${\varphi }_0$ is the magnitude of the incident potential field; ${J}_m$ and $H_m^{( 1 )}$ are the Bessel and Hankel functions of the first kind with order *m*, standing for the incident and scattering waves, respectively; ${k}_4$ is the wave number in the region IV; and ${S}_m$ is the scattering coefficient of the *m*-th angular momentum channel. We determine ${S}_m$ by applying the continuity conditions of the potential energy field and the flow. The flow *F* can be written as $F = u( {\nabla \varphi } )$ and $u = tanh ( {kh} )/k$ [19] (see Supplementary materials for details). The total SCS, i.e. the total scattered power over the intensity of the incident waves, can be expressed by ${C}_{sct} = \mathop \sum \nolimits_{m = - \infty }^\infty {C}_{sct,m} $, where ${C}_{sct,m} = ( {2\lambda /\pi } )|{S}_m{|}^2$. Since $|{S}_m{|}^2 \le 1$, the SCS from the individual channel is bound by the single-channel scattering limit, i.e. ${C}_{sct,m} \le 2\lambda /\pi $.

By virtue of the simulated annealing algorithm [42], we optimize the geometries and working frequencies of a superscatterer, including heights and radii (see Supplementary Note 2 for design examples). Due to the constraints of the experiment set-up (the frequency of the wave maker) and to closely mimic the frequencies of natural water waves, a set of parameters are ultimately chosen. The radii are ${r}_1 = \ 0.0931\ {\rm{m}}$, ${r}_2 = \ 0.0938\ {\rm{m\ and}}$${r}_3 = \ 0.1950\ {\rm{m}}$; the heights of the cylinders are ${h}_1 = \ 0.1574\ {\rm{m}}$, ${h}_2 = \ 0.0701\ {\rm{m\ and}}$${h}_3 = \ 0.1453\ {\rm{m}}$; the depth of the simulated range is $d = 0.16\ {\rm{m}}$; and the working frequency is $f = 1.55{\rm{\ Hz}}$ (${\rm{\lambda \ }} = 0.81\ {\rm{m}}$). In this way, the diameter of the designed superscatterer only has 0.48 times of the working wavelength. Figure 1b shows the analytical SCS of the superscatterer, where the SCS peaks from $m = \pm 2$, $m = \pm $3 angular momentum channels are almost overlapped. The total SCS far exceeds the single-channel scattering limits (3.367 times). For comparison, we also analyse the scattering of a uniform cylinder with the same radius, termed as a trivial scatterer ($r = 0.1950\ {\rm{m}},h = 0.1574\ {\rm{m}}$). In stark contrast, the total SCS is far smaller than the single-channel scattering limit (0.129 times), as illustrated in Fig. 1d. For trivial scatterers, those angular momentum channels that do not support resonances usually contribute little to the total SCS. Therefore, if the resonances are separated, the total SCS is constrained by the single-channel limit.

## NUMERICAL SIMULATION

We perform numerical simulation to illustrate the performance of the designed superscatterer using the commercial COMSOL Multiphysics solver package. The computational domain (8 × 8 m) is truncated by perfectly matched layer absorbing boundaries and the plane water wave is incident from the left to right side. We set all the parameters in the simulation, including the size of the superscatterer, the tank size and the initial water depth. Then, we used the PDE interface to simulate the water wave by solving the equation$\ \nabla \cdot ( {u\nabla \varphi } ) + ( {{\omega }^2/g} )\varphi $, which is analogous to the Helmholtz equations for EM waves.

**Figure 2.**
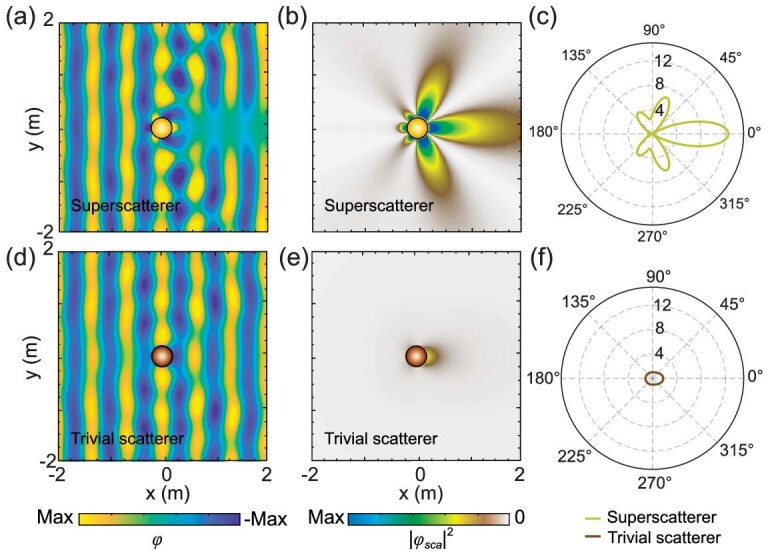
Analysis and simulation results of water-wave superscattering. (a and d) Simulated total field pattern of the superscatterer and the trivial scatterer. (b and e) Scattering energy created by the superscatterer and the trivial scatterer. The plane waves are incident from the left to right side. The superscatterer and the trivial scatterer are highlighted by yellow and gray circles, respectively. (c and f) Scattering cross section per azimuth angle for the superscatterer and the trivial scatterer. The radial length represents the magnitude of cross section per azimuthal angle, which is in units of $2\lambda /\pi .$

Figure 2a and d illustrate the simulated field patterns of the water waves for the superscatterer and the trivial scatterer at the working frequency. The water wave is incident from the left to right as a plane wave. For simplicity, we zoom in on the central 2 × 2 m region. When the water wave passes through the superscatterer, a huge ‘shadow’ is produced in the forward direction, while the water wave is enhanced near the superscatterer. In contrast, for the trivial scatterer, the water wave does not change drastically before and after passing through it. In order to observe the superscattering effect more clearly, we plot the scattered field $(|{\varphi }_{sca}{|}^2)\ $in Fig. 2b and e. There are considerable differences between the scattering strength caused by the superscatterer and the trivial scatterer. Figure 2c and f illustrates the SCS per azimuthal angle (i.e. the scattering width), calculated by using ${C}_{sw} = \ ( {2\lambda /\pi } ){| {\mathop \sum \nolimits_{m = - \infty }^\infty {S}_m{\rm{cos}}( {m\phi } )} |}^2$ [44]. The relationship between the total SCS and the scattering width is ${C}_{cst} = \ ( {1/2\pi } )\mathop \smallint \nolimits_0^{2\pi } {C}_{sw}d\phi $. Evidently, the scattered waves from the superscatterer mainly appear in the forward direction and are much stronger than that of the trivial scatterer.

## EXPERIMENTAL OBSERVATION

**Figure 3.**
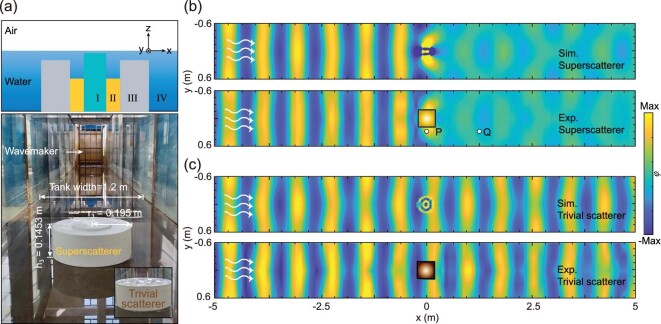
Experimental observation of superscattering. (a) Schematic of the fabricated superscatterer. (b) Experimental set-up of the superscatterer. The inset shows a uniform cylinder with the same size. The experiment was carried out in a (60 × 1.2 × 2 m) glass tank. At the front, a wave maker generates plane water waves to propagate along the water tank, which are absorbed by absorbing material at the end. (b and c) Measured and simulated field patterns of the superscatterer and the trivial scatterer. The square area in the vicinity of the scatterer was not measured due to the experimental inconvenience. The white arrow represents the propagation direction. At 1.55 Hz, the superscatterer has a greater scattering effect, in stark contrast to that of the uniform cylinder. The simulated and experimental results are in good agreement. The points *P* and *Q* marked in (b) represent the points that we will focus on in the following experiment.

We fabricated the superscatterer using the 3D printing technique to observe the superscattering phenomenon in an experiment. Figure 3a shows the schematic of the water-wave superscatterer; the whole area is divided into four regions with different water depths along the *z*-direction. In principle, the experiment requires a much larger range and a large water-wave generator to create the incident plane waves. However, due to the limited experimental space, the experimental sample was designed to be applied under a hard boundary. We chose a glass tank (60 × 1.2 × 2 m), i.e. a hard boundary, instead of an absorbing boundary; then we also performed simulation under this boundary condition (in Fig. 3b and c). More experimental photographs and measurement details appear in the Supplementary materials.

The superscatterer is flanked by the rigid glass of the water tank, with a wave maker placed at the head and a wave-absorbing material at the tail. The bottom was fixed in the center of the water tank using glue. In the experiment, we varied the water depth in the water tank from 0.15 to 0.17 m for exploring various phenomena. The front side of the tank was set up by the wave maker, which could generate water waves with different amplitudes, ranging from 0.2 to 2 Hz, and the tail of the tank was installed with a wave-absorption device. We use the linear wave incidence condition, where the wave height is <1/50 of the wavelength, to control the wave maker to generate linear water waves. We try to control all parameters to meet the linearity condition and wait for the waveform created by the wave maker to reach a stable linear wave before starting the measurement to avoid the non-linear effect of water waves. It should be noted that our structure is set at a distance of 15 m from the wave maker at the front of the tank, with a measurement area of 10 m and a distance of 45 m from the wave absorber at the end of the tank. The time from the wave maker to the wave absorber is ∼50 s, which is long enough. The problem of water-wave dissipation due to viscosity and the effect of reflected waves from the absorber are eliminated as far as possible. We measure the wave amplitude by placing the water-wave sensor in different positions in the water tank. The wave-field diagram was generated based on the measured data of the wave amplitude (Supplementary materials).

Figure 3b and c shows the measured field patterns of the superscatterer and the trivial scatterer, as well as the simulated results. The light yellow and gray squares in Fig. 3b and c represent the locations of the superscatterer and trivial scatterer, respectively. We measured the field diagrams with the frequency from 1.53 to 1.57 Hz and the water depth from 0.15 to 0.17 m, and the superscattering works well at 1.55 Hz and 0.16 m. The amplitude of the water wave near the superscatterer is enhanced and then is weakened in the forward propagation. However, when the water wave passes through the trivial scatterer, its waveform remains almost unchanged. To be consistent with the experimental set-up, we also adjust the boundary on both sides in the simulation, which could help us to explore the effect of different boundaries on the water-wave superscattering phenomenon. The simulated results are similar to the situations with the absorption boundary in Fig. 2.

**Figure 4.**
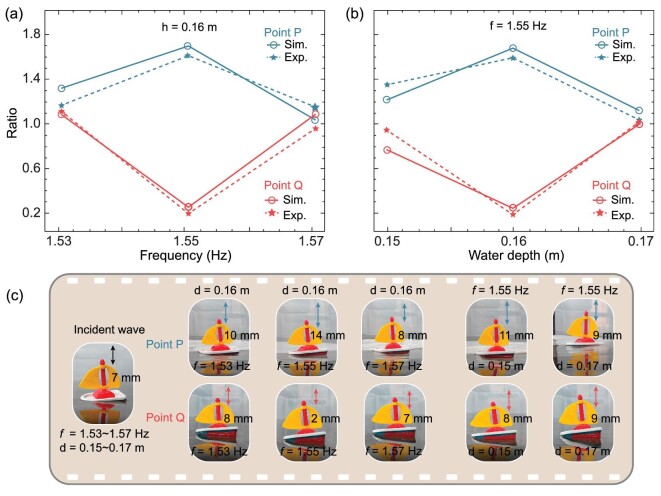
Visualization of superscattering effects at different frequencies and water depths. The vertical displacement of the plastic boat was characterized as the amplitude of the incident wave. The data were measured by using a wave sensor. We measured the vertical displacements at points *P* (around the superscatterer) and *Q* (at the rear of the structure) separately, which are normalized by the incident amplitude to obtain the ratios. (a) Amplitude ratio versus frequency. (b) Amplitude ratio versus water depth. (c) Screenshots from experimental videos. The vertical displacement at point *P* is much larger than that at point *Q*, especially at the designed working frequency (1.55 Hz) and water depth (0.16 m).

To visualize the superscattering effect, we placed a tiny plastic boat at points *P* and *Q*, as marked in Fig. 3b. Point *P* is located close to the superscatterer and point *Q* is 1.25 m away from point *P*. The amplitude of the water waves can be reflected by the vertical motion of the plastic boat, while its horizontal motion is confined by ropes fixed to the tank bottom. We first measure the amplitude of the incident water waves without any sample, as shown in the inset of Fig. 4c. Throughout the experiment, the amplitude of the incident water waves remained almost unchanged, i.e. ${A}_{inc} = \ 7\ {\rm{mm}}$. The enhancement or suppression of the amplitudes at points *P* and *Q* can be characterized by the ratios ${A}_P/{A}_{inc}$ and ${A}_Q/{A}_{inc}$, respectively. Specifically, we consider two situations. First, the water depth is fixed at $d = 0.16{\rm{\ m}}$ and the incident frequency is varied from 1.53 to 1.57 Hz, as shown in Fig. 4a and the left panel of Fig. 4c. Second, the incident frequency is fixed at 1.55 Hz, while the water depth ranges from 0.15 to 0.17 m, as shown in Fig. 4b and the right panel of Fig. 4c. Also, we attached the raw experimental videos to show the dynamics of the tiny boat in the Supplementary materials. The experimental and simulated results are consistent in general. The amplitude at point *P* is the highest, especially at the designed working frequency (1.55 Hz) and water depth (0.16 m). This may benefit some applications in energy harvesting, such as high-efficiency mechanical generators. In contrast, the amplitude at point *Q* is very slight because the incident and scattered waves are out of phase (destructive interferences) in the forward scattering. This is useful for creating superscatterer-based miniaturized breakwaters for the applications of harbor protection.

## CONCLUSIONS

In conclusion, we have theoretically predicted and experimentally observed the superscattering phenomenon in water waves. The underlying mechanism originates from the resonances of different angular moment channels. The superscatterer found here provides a facile yet viable way to significantly enhance water-wave scattering by simply designing an inhomogeneous depth profile on the bottom. The experimental results are well confirmed by the near-field patterns, which are consistent with theoretical analysis and numerical simulation. Our method has important applications, e.g. the strong fluctuation near the superscatterer can be used for energy harvesting and the large shadow can be used for harbor protection. Looking forward, we envisage that structural variants combined with machine learning can realize an adaptive superscattering for different frequencies and water depths or multi-frequency superscattering [45,46]. Further realistic effects, such as fluid viscosity and wave nonlinearity, should also be included [47,48]. The continuous exploration of such exotic phenomena in water waves is highly desired, since it not only refreshes the physical understanding, but also extends the capability to manipulate water waves for off-the-shelf applications [2,22,23,28–32].

## Supplementary Material

nwac255_Supplemental_Files
