# Supplementary material for: Superscattering of water waves
Source: Natl Sci Rev. 2022 Nov 10;10(7):nwac255. doi: 10.1093/nsr/nwac255 (PMC10232047; doi:10.1093/nsr/nwac255)
Supplement: nwac255_Supplemental_Files [file nwac255_supplemental_files.zip › Supplementary Materials.docx]

Supplementary Information for

**Superscattering of water waves**

Supplementary note 1: scattering cross section

As a conceptual demonstration, below we analytically calculate the scattering cross section of a 2D water wave scattering from a sub-wavelength multilayer rod surrounded by water. The multilayer rod is shown in the inset of Fig. 1(a). We consider linear, inviscid, and irrotational water waves in infinite extent of water with a constant depth $d$. The multilayer rod is composed of three concentric cylinders of different heights $h_{1}\mathrm{to} h_{3}$ and radii $r_{1}\mathrm{to}r_{3}$. The three different heights segment the water surface height into four different heights${d-h}_{1},{d-h}_{2},{d-h}_{3}$ and $d$. We assume that the incident water wave propagates along the $+x$ direction. By using the cylindrical coordination $\left( \rho,\text{ϕ} \right)$ with the origin at the centre of the cylinder, $\varphi$ for each region can be written as:

$\varphi_{1}=\sum_{m} A_{m}J_{m}(k_{1}\rho)e^{im\text{ϕ}} \rho\leq r_{1}$(1)

$\varphi_{2}=\sum_{m} \left[ B_{m}J_{m}(k_{2}\rho)+C_{m}H_{m}(k_{2}\rho) \right]e^{im\text{ϕ}} r_{1}\leq\rho\leq r_{2}$(2)

$\varphi_{3}=\sum_{m} \left[ D_{m}J_{m}(k_{3}\rho)+E_{m}H_{m}(k_{3}\rho) \right]e^{im\text{ϕ}} r_{2}\leq\rho{\leq r}_{3}$ (3)

$\varphi_{4}=\sum_{m} \left[ F_{m}J_{m}(k_{4}\rho)+H_{m}(k_{4}\rho) \right]e^{im\text{ϕ}} \rho\geq r_{3}$ (4)

Herein, $J_{m}$ and $H_{m}$ are the $m$th order of Bessel function and Hankel function. $k_{1}$ to$k_{4}$ are the wave numbers in regions I to IV, respectively. $\rho$ are the radius of each level of the structure. The flow $F=u\nabla\varphi$ and reduced depth $u$ can be obtained from previous study [[1](#_ENREF_1)]. $F$ for each region can be written as:

$F_{1}=u_{1}k_{1}\sum_{m} A_{m}J'_{m}(k_{1}\rho)e^{im\text{ϕ}} \rho\leq r_{1}$ (5)

$F_{2}=u_{2}k_{2}\sum_{m} \left[ B_{m}J'_{m}(k_{2}\rho)+C_{m}H'_{m}(k_{2}\rho) \right]e^{im\text{ϕ}} r_{1}\leq\rho\leq r_{2}$ (6)

$F_{3}=u_{3}k_{3}\sum_{m} \left[ D_{m}J'_{m}(k_{3}\rho)+E_{m}H'_{m}(k_{3}\rho) \right]e^{im\text{ϕ}} r_{2}\leq\rho{\leq r}_{3}$ (7)

$F_{4}=u_{4}k_{4}\sum_{m} \left[ F_{m}J'_{m}(k_{4}\rho)+H'_{m}(k_{4}\rho\right]e^{im\text{ϕ}} \rho\geq r_{3}$ (8)

where the reduced depth satisfies $u_{j}=\frac{\left[ \tanh(k_{j}h_{j}) \right]}{k_{j}}, j=1,2,3,4$. At the boundary of the water column, the potential $\varphi$ and flow$F=u\nabla\varphi$ should be continuous. By matching the boundary conditions $\varphi$ and $F$ for each boundary, we obtain a series of equations as below

At $\rho\text{= }r_{3}\text{ }\varphi_{3}=\varphi_{4} F_{3}\text{=}F_{4}$

$D_{m}J_{m}\left( k_{3}d_{3} \right)+E_{m}H_{m}\left( k_{3}d_{3} \right)=F_{m}H_{m}\left( k_{3}d_{3} \right)+J_{m}\left( k_{4}d_{3} \right)$ (9)$u_{3}k_{3}\left[ D_{m}J'_{m}\left( k_{3}d_{3} \right)+E_{m}H'_{m}\left( k_{3}d_{3} \right) \right]=u_{3}k_{3}\left[ F_{m}H'_{m}\left( k_{3}d_{3} \right)+J'_{m}\left( k_{3}d_{3} \right) \right]$ (10)

At $\rho\text{=}r_{2}\text{ }\varphi_{2}=\varphi_{3} F_{2}\text{ = }F_{3}$

$B_{m}J_{m}\left( k_{2}d_{2} \right)+C_{m}H_{m}\left( k_{2}d_{2} \right)=D_{m}J_{m}\left( k_{3}d_{2} \right)+E_{m}H_{m}\left( k_{3}d_{2} \right)$ (11)$u_{2}k_{2}\left[ B_{m}J'_{m}\left( k_{2}d_{2} \right)+C_{m}H'_{m}\left( k_{2}d_{2} \right) \right]=u_{3}k_{3}\left[ D_{m}J'_{m}\left( k_{3}d_{2} \right)+E_{m}H'_{m}\left( k_{3}d_{2} \right) \right]$ (12)

At $\rho\text{= }r_{1}\text{ }\varphi_{1}=\varphi_{2} F_{1}\text{= }F_{2}$

$A_{m}J_{m}(k_{1}d_{1})=B_{m}J_{m}\left( k_{2}d_{1} \right)+C_{m}H_{m}\left( k_{2}d_{1} \right)$ (13)

$u_{1}k_{1}[A_{m}J'_{m}\left( k_{1}d_{1} \right)]=u_{2}k_{2}\left[ B_{m}J'_{m}\left( k_{2}d_{1} \right)+C_{m}H'_{m}\left( k_{2}d_{1} \right) \right]$ (14)

$A_{m}$ to $F_{m}$ are unknown scattering coefficients and can be determined through the above boundary conditions. The six linear equations can exactly determine the six factors. By solving the boundary conditions, the total potential field in the water region can be expressed as:

$\varphi_{total}=\varphi_{0}\sum_{m=-\infty}^{\infty} \left( i^{m}J_{m}\left( k_{4}\rho\right)e^{im\phi}+i^{m}S_{m}H_{m}^{(1)}\left( k_{4}\rho\right)e^{im\phi} \right)$ (15)

where $S_{m}$is the scattering coefficient for the $m^{th}$ angular momentum channel; $\varphi_{0}$ is the magnitude of incident potential field. The total scattering cross section can be expressed as $C_{sct}=\sum_{m=-\infty}^{\infty} C_{sct,m}$ ${C_{sct,m}= \frac{2\lambda}{\pi}|S_{m}|}^{2}$ .

Supplementary note 2: more superscatterer design

The simulated annealing algorithm is adopted to optimize the multilayer structure to realize the phenomenon of superscattering. The detailed flow-process is indicated below.

Target: The aim is to maximize the total scattering cross section $C_{sct}$ from channels |𝑚| = 1,2,3. This means that the scattering cross section of each individual channel |𝑚| = 1,2,3 is reached as close as possible to the single channel limit. In addition, the design of the superscatterer should be sub-wavelength in size and satisfy the linear water wave condition

Variables: There are four free variables, including the height and radius for each region, the frequency and water depth.

Flow-process:

1. Initialize the temperature 𝑇 (a self-defined parameter for the simulated annealing algorithm), iteration number 𝐿 and solution 𝑆 (see Eq. (S15)). Then calculate the scattering cross section $C_{sct}$.

2. Generate a new solution $S^{'}$, calculate the scattering cross section $C_{sct}(S^{'})$ with the increment $\Delta T{=C}_{sct}\left( S^{'} \right)-C_{sct}(S)$.

3. Determine whether we shall accept the new solution $S^{'}$, based on the Metropolis rule. To be specific, if $\Delta T>0$, we accept the new solution $S^{'}$. Otherwise, we accept it with a probability $exp(-\Delta T/T)$.

4. Execute step 2 and 3 for $L$ times. If the target is achieved during the iteration, the optimization ends.

5. Decrease the temperature $T$ and then repeat steps 2 to 4.

By virtue of simulated annealing algorithm, the subwavelength structure having superscattering ability is achieved. We have designed many superscatterers with different geometries and working frequencies. On account of the constraints in experimental setup, and to more closely approximate the natural water waves, we ultimately choose the structure in Fig. 1 of the main text (although the total SCS created by the structure is not the optimal).

In Fig. S1(a), the radii are $r_{1}=0.1330 m$, $r_{2}=0.1436 m$, $r_{3}=0.1872 m$, the heights of cylinder are $h_{1}=0.1345 m$ , $h_{2}=0.1595 m$ , $h_{3}=0.1466 m$, the depth of simulated range is $d=0.16 m$. At the working frequency $f=1.99 Hz$ ($\lambda=0.62 m$), the SCS peaks from $m=\pm1$, $m=\pm2,m=\pm$3 angular momentum channels are almost overlapped, yielding the total SCS far beyond the single-channel scattering limits (5.86 times).

In Fig. S1(b), the radii are $r_{1}=0.0778 m$, $r_{2}=0.1526 m$, $r_{3}=0.1895 m$, the heights of cylinder are $h_{1}=0.0507 m$ , $h_{2}=0.1573 m$ , $h_{3}=0.1059 m$, the depth of simulated range is $d=0.16 m$. At the working frequency $f=2.96 Hz$ ($\lambda=0.42 m$), the total SCS far exceeds the single-channel scattering limits (5.88 times).

In Fig. S1(c), the radii are $r_{1}=0.1027 m$, $r_{2}=0.1314 m$, $r_{3}=0.1947 m$, the heights of cylinder are $h_{1}=0.1597 m$ , $h_{2}=0.1569 m$ , $h_{3}=0.1273 m$, the depth of simulated range is $d=0.16 m$. At the working frequency $f_{1}=2.26 Hz$ ($\lambda_{1}=0.55 m$), $f_{2}=2.48 Hz$ ($\lambda_{2}=0.50 m$), this structure enables multi-frequency superscattering phenomenon and the total SCS is 5.72 and 5.61 times of the single-channel scattering limits. It is worth noting that the above results realize a broadband superscattering to different extents, which means that the scattering intensity is greater than the trivial scatterer at a certain frequency range. This also demonstrates that through proper inverse design, superscatterers can realize broadband.


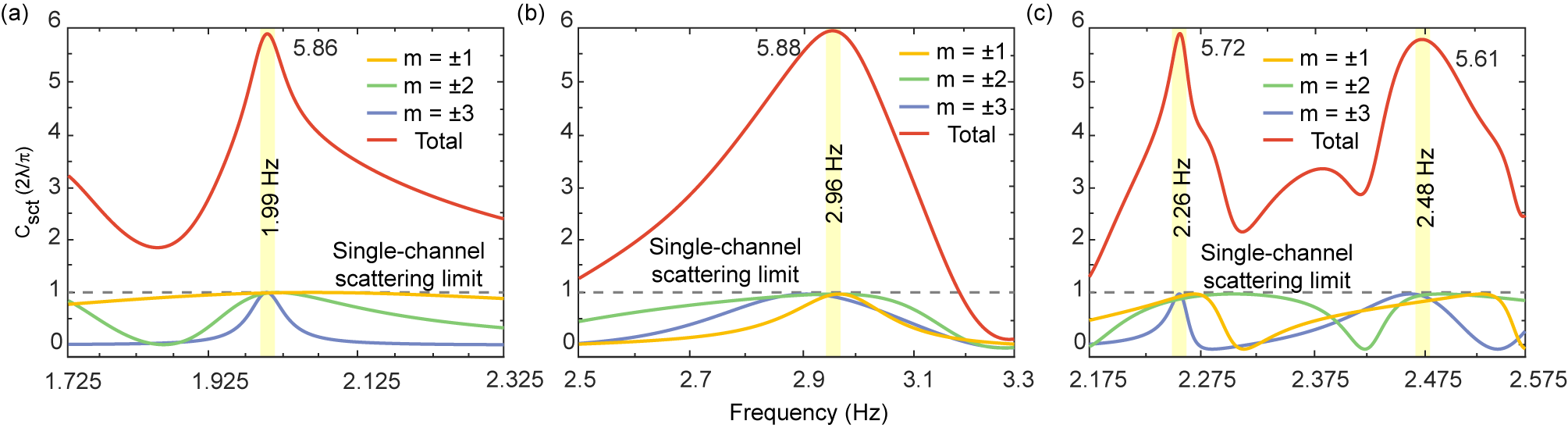


**Figure S1**. More superscatterer designs.

Supplementary note 3: experimental measurements

The experiments were carried out in a water tank (60 m*1.2 m*2 m), with a wave maker upstream and a wave absorber downstream as shown in Fig. S2. The water tank is covered with glass walls on both sides and a horizontal concrete floor at the bottom. Because the waveforms were symmetrically distributed along the centre line of the tank, only half of the measurements were required. As shown in Fig. S3, three wave gauges were used in the experiment, with measurements taken at intervals of approximately half a wavelength (0.4 m), and the gauges were tied up with wooden strips to ensure accuracy when moving horizontally.


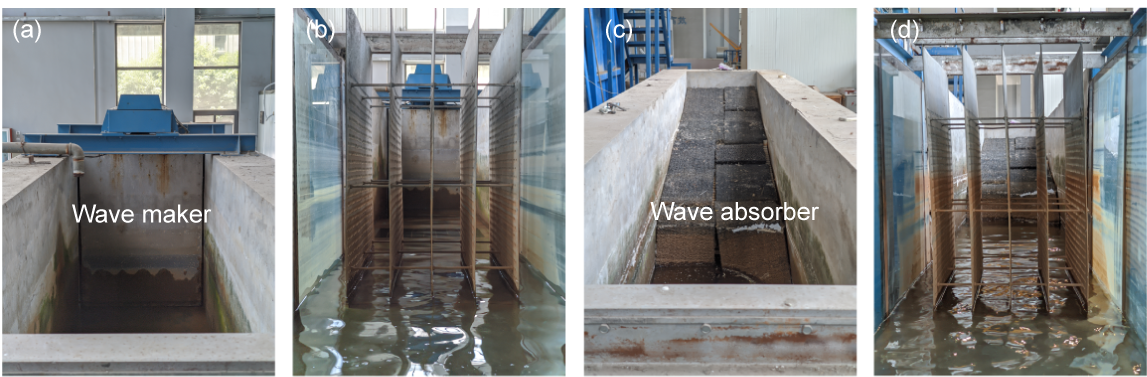


**Figure S2**. Wave maker and wave absorber.


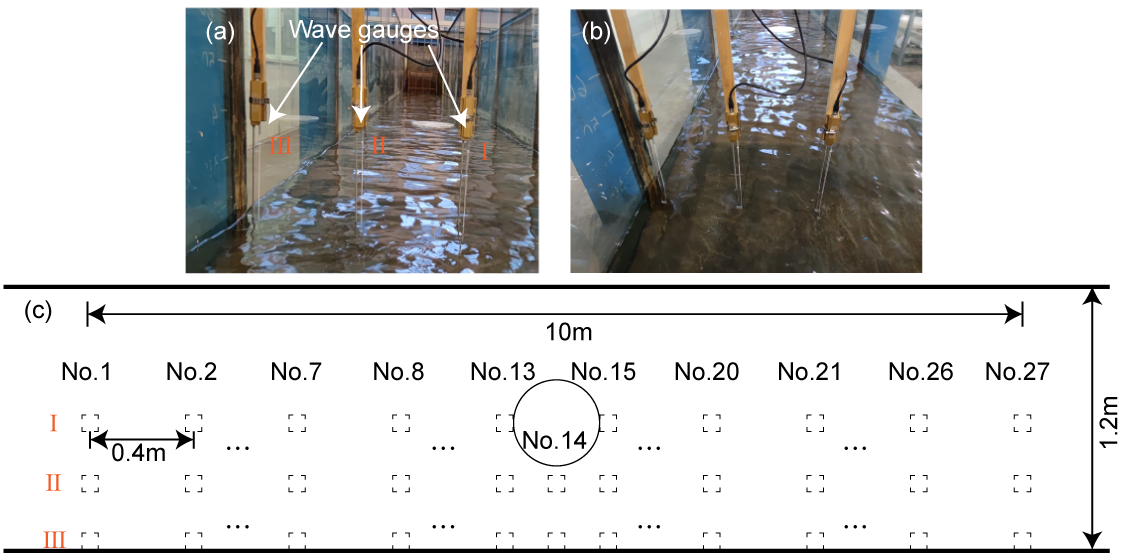


**Figure S3**. Wave gauges and measurement methods.

Supplementary note 4: water wave experiment video

To visualize the effect of water wave superscattering, a model boat was placed at two different locations, points $P$ and $Q$, as marked in Fig. 3(b). The amplitude of water waves can be reflected by the vertical motion of the plastic boat, while its horizontal motion is confined by ropes fixed to the tank bottom. We measured water depth from $d =0.15$to$0.17 m$. For the frequencies, we have chosen from $f=1.53$to $1.57 Hz$ respectively. We write the amplitude of points $P$, $Q$ separately as$A_{P}$ $A_{Q}$ . And the amplitude of Incidence is almost identical, about$A_{inc}=7 mm$.

The following are videos we recorded of the movement of the model boat:

- Supplementary Video S1 (.mp4 format). Comparison of superscatterer and trivial scatterer of model boat motion when water depth is 0.16 m and frequency is 1.55 Hz.
- Supplementary Video S2 (.mp4 format). Comparison of model boat motion when water depth is 0.16 m and frequency is from 1.53 to 1.57 Hz at point $P$.
- Supplementary Video S3 (.mp4 format). Comparison of model boat motion when frequency is 1.55 Hz and water depth is from 0.15 m to 0.17 m at point $P$.
- Supplementary Video S4 (.mp4 format). Comparison of model boat motion when water depth is 0.16 m and frequency is from 1.53 to 1.57 Hz at point $Q$.
- Supplementary Video S5 (.mp4 format). Comparison of model boat motion when frequency is 1.55 Hz and water depth is from 0.15 m to 0.17 m at point $Q$.

REFERENCES

1. Hu X, Chan CT, Ho KM *et al.* Negative effective gravity in water waves by periodic resonator arrays. *Phys Rev Lett* 2011; **106**: 174501.
